# Post‐COVID‐19 Disorders of Gut‐Brain Interaction: A New Challenge for Gastroenterologists

**DOI:** 10.1002/ueg2.70008

**Published:** 2025-03-05

**Authors:** Mohamed G. Shiha, Imran Aziz

**Affiliations:** ^1^ Division of Clinical Medicine School of Medicine and Population Health University of Sheffield Sheffield UK; ^2^ Department of Gastroenterology University Hospitals of Leicester Leicester UK; ^3^ Academic Unit of Gastroenterology Sheffield Teaching Hospitals Sheffield UK

Post‐infectious disorders of gut‐brain interaction (DGBI) are well‐recognised consequences of gastrointestinal infections and may persist for years after the initial illness [1]. This persistence is thought to be driven by long‐term neural sensitisation and pro‐nociceptive changes in the gut micro‐environment, leading to altered pain perception and gut function in the absence of detectable tissue inflammation [2]. SARS‐CoV‐2, the coronavirus that causes COVID‐19, shares a similar potential for triggering gut dysfunction due to its unusual high affinity for human angiotensin‐converting enzyme‐2 receptors, which are abundantly expressed in the gastrointestinal tract [3]. Studies have shown that patients with COVID‐19 have a significantly increased risk of developing DGBI compared with non‐infected controls [4, 5]. A recent meta‐analysis reported that 12% of patients developed irritable bowel syndrome (IBS) and 4% developed functional dyspepsia after COVID‐19, with the risk being about six times higher for IBS and eight times higher for functional dyspepsia compared with controls [4]. Given the vast number of COVID‐19 survivors globally, even a modest increase in the incidence of DGBI could result in a significant healthcare burden.

In this issue of *United European Gastroenterology Journal*, Marasco, Hod, and colleagues provide longitudinal data from an international multicentre prospective study on the progression of gastrointestinal symptoms and psychological distress amongst 599 patients hospitalised with COVID‐19 infection [6]. Participants were categorised as those with post‐COVID‐19 DGBI (4.5%), pre‐existing DGIB (10.2%), and non‐DGBI controls (85.3%). Over the course of a year, patients completed a series of standardised questionnaires at multiple time points following discharge. The authors found that patients who developed post‐COVID‐19 DGBI experienced progressive worsening of gastrointestinal symptoms and no improvement in psychological distress. In contrast, those with pre‐existing DGBI and non‐DGBI controls showed gradual recovery in most gastrointestinal and psychological symptoms after 1 year from the acute infection. These findings are consistent with growing evidence that COVID‐19 significantly increases the risk of developing DGBI and contributes to persistent gastrointestinal symptoms, particularly in those who experienced gastrointestinal symptoms during the acute infection or developed psychological trauma related to the hospitalisation [7, 8].

How do these results align with our current understanding of the prognosis of post‐infectious DGBI? A 2017 meta‐analysis found that the risk of IBS after viral enteritis is highest within the first year but declines thereafter, eventually becoming comparable to that of the general population [9]. However, a recent study showed that the prevalence of post‐COVID‐19 DGBI was significantly higher than in the general population more than 1 year after hospitalisation [8]. This suggests that post‐COVID‐19 DGBI could represent a distinct entity with a more protracted disease course than traditional post‐infectious DGBI. Further studies with extended follow‐up are needed to clarify the long‐term prevalence and trajectory of post‐COVID‐19 DGBI. In the meantime, family practitioners and gastroenterologists should prepare for the growing burden of post‐COVID‐19 DGBI, as these patients are likely to have more frequent clinical visits, undergo additional diagnostic testing and contribute to increased healthcare utilisation.

The current study highlights the need for a better understanding of the underlying pathophysiological mechanisms driving symptom persistence or resolution in patients with post‐COVID‐19 DGBI. Equally important is evaluating the clinical and mechanistic role of therapies targeting the microbiome‐brain‐gut axis, such as dietary modifications, probiotics, neuromodulators and behavioural interventions [10]. This remains an area of unmet need in post‐infectious DGBI, and with the emergence of post‐COVID‐19 DGBI, it is hoped that it will become a research funding priority to improve patient outcomes.

## Conflicts of Interest

M.G.S. is a Trainee Editor at UEG Journal. I.A. received speaker fees from PrecisionBiotics.

## Data Availability

Data sharing is not applicable to this article as no new data were created or analyzed.
